# A Meta-Analysis of Endoscopic vs. Microscopic Transsphenoidal Surgery for Non-functioning and Functioning Pituitary Adenomas: Comparisons of Efficacy and Safety

**DOI:** 10.3389/fneur.2021.614382

**Published:** 2021-03-23

**Authors:** Shengfu Guo, Zidong Wang, Xiaokui Kang, Wenqiang Xin, Xin Li

**Affiliations:** ^1^Department of Neurosurgery, Liaocheng People's Hospital, Liaocheng, China; ^2^Department of Neurology, University Medical Center Goettingen, Göttingen, Germany; ^3^Department of Neurosurgery, Tianjin Medical University General Hospital, Tianjin, China

**Keywords:** pituitary adenoma, non-functioning, functioning, endoscopic, microscopic

## Abstract

**Background:** Although microscopic (MTSS) and endoscopic transsphenoidal surgery (ETSS) are both effective approaches for treating non-functioning pituitary adenomas (NFPA) and functioning pituitary adenomas (FPA), the consensus remains unidentified on whether there are differences in the risk of postoperative complications between the two surgical approaches.

**Method:** A meta-analysis of the study of MTSS vs. ETSS for NFPA and FPA was conducted by searching the electronic databases of PubMed, Cochrane Library, and EMBASE, from the date of establishment of electronic databases to September 2020 based on the PRISMA guidelines.

**Results:** In this study, a total of 16 studies were selected, hailing from Belgium, the USA, India, Finland, France, Korea, Spain, China, and Canada. We enrolled 1003 patients in the ETSS and 992 patients in the MTSS group. In patients with NFPA, the ETSS group was related to a higher incidence of post-operative gross-total resection (GTR). (OR = 1.655, 95% CI 1.131–2.421, *P* = 0.010). In participants with FPA, the results illustrated that the ETSS group had higher rates of visual improvement (OR = 2.461, 95% CI 1.109–5.459) and gross-total resection (OR = 2.033, 95% CI 1.335–3.096), as well as lower meningitis rates (OR = 0.195, 95% CI 0.041–1.923). In participants with acromegaly, no significant difference was shown in the postoperative complications.

**Conclusion:** Based on current evidence, participants with NFPA treated by endoscopy were related to higher rates of GTR; patients with FPA treated by ETSS were related to higher rates of visual improvement and GTR, as well as a lower rates of meningitis.

## Introduction

Pituitary adenomas, which contribute to 14% of primary intracranial neoplasms, are the second most common central nervous system tumor. The incidence of pituitary adenomas in the general population has increased to 17% (1, 2). 36–54% of pituitary adenomas are non-functional, while 46–64% of them are hormone-secreting (3–5). Even though a large proportion of pituitary adenomas are histologically benign, due to their location being close to crucial structures and the hypersecretion or paracrisis of the pituitary hormone, they can result in serious endocrine conditions, such as acromegaly and Cushing disease (6).

In the 1960s, Hardy (7) reported an operative microscope for better visualization during transsphenoidal surgery, which enabled the safer removal of sellar tumors. Microscopic transsphenoidal pituitary surgery (MTSS) had at the time been widely performed and became the gold standard. But within the next few years, and with the evolution of endoscopic techniques, by 1992, Jankowski et al. (8) performed fully endoscopic surgery *via* the endonasal approach for pituitary tumors. Since then, endoscopic transsphenoidal pituitary surgery (ETSS) has increasingly been adopted. Nevertheless, establishing which surgical method is superior in managing pituitary tumors, remains unknown. ETSS enjoys more popularity, attributed to the panoramic view of its surrounding structures and minimal invasiveness, leading to a greater chance of removing central skull base lesions (9, 10). Whereas, ETSS has limitations of two-dimensioned visualization, less focus capacity, and a steep learning curve for neurosurgeons. Furthermore, consensus on whether there are differences in the risk of postoperative complications between the two surgical approaches remains unclear (11).

Recently, several studies on ETSS vs. MTSS has been reported in the literature. We thus set out to perform a meta-analysis to evaluate the postoperative outcomes between ETSS and MTSS, in participants with non-functioning pituitary adenomas (NFPA) or functioning pituitary adenomas (FPA).

## Materials and Methods

The systematic review and meta-analysis of the literature was performed based on the Preferred Reporting Items for Systematic Reviews and Meta-Analyses (PRISMA) (12).

### Literature Search

The search object was research literature on the analysis of all comparative studies of ETSS vs. MTSS for non-functioning and functioning pituitary adenomas published in publicly available electronic databases, including PubMed, Cochrane Library, and EMBASE, from the date of establishment of electronic databases to September 2020. To obtain maximum results in identifying relevant literature, the following literature search keywords were adopted: “transsphenoidal surgery,” “pituitary,” “non-functioning,” “functioning,” “endoscopic,” “microsurgical,” and “acromegaly.” The specific search strategy for publications comparing endoscopic vs. microscopic transsphenoidal surgery is shown in Table 1. Furthermore, the reference lists of relevant studies and reviews were manually checked by two authors, to further identify other potential studies in the literature.

**Table 1:** The search strategy for studies comparing endoscopic vs. microscopic transsphenoidal surgery.

Transsphenoidal surgery OR Neurologic surgical procedure OR Neuroendoscopy OR Microsurgery
**AND**
Pituitary OR Pituitary and surgery OR Pituitary adenomas OR Pituitary neoplasm
**AND**
(Non-functioning OR Functioning
**OR**
Acromegaly OR GH-secreting adenoma OR GH-producing adenoma OR Somatotroph tumor)

### Inclusion and Exclusion Criteria

The study selection was in accordance with the following PICOS criteria: (I) population: strictly refers to the NFPA or FPA; (II) intervention: ETSS and MTSS; (III) comparison: the outcomes of procedure-related efficacy and safety; (IV) outcome measures: reports one or more of the including endpoints: gross-total resection (GTR), cerebrospinal fluid (CSF) leak, length of stay, visual improvement, hypothyroidism, meningitis, hematoma, operation time, diabetes insipidus, hypopituitarism, hypocortisolism, and mortality; and (V) the full-text of publications were written in English.

Our exclusion criteria are: (I) studies that lack details of postoperative efficacy or complications; (II) studies with an imbalance of clinical characteristics; (III) non-investigative studies like case report, case series, and single-armed studies; (III) conference proceedings, letters, animal trials, systematic reviews, and meta-analyses.

### Data Extraction

We assigned three authors to extract information of included studies, respectively. Two authors extracted all study design details from the full text of the included study, then the third author checked all extracted data. Discrepancies were resolved by consulting clinical experts. For each included study, the following details were extracted: study characteristics (first author, year of publication, country, sample size, study type), case characteristics (sex and age), and operation type. The following outcome items were also extracted: GTR, length of stay, diabetes insipidus, visual improvement, CSF leak, hypothyroidism, hypopituitarism, meningitis, hematoma, hypocortisolism, operation time, and mortality.

### Statistical Analysis

Comparisons of the postoperative complications after ETSS and MTSS were analyzed by the standard software STATA version 12.0. Heterogeneity among studies was assessed using Cochran's *Q*-test (*p* < 0.10) and the *I*^2^-value. In brief, when the *I*^2^ > 50%, we considered the heterogeneity to be high, and a random-effect model would be selected. Otherwise, a fixed-effect model was selected. As for continued variables, the length of stay and operation time were expressed as weighted mean differences (WMD) with 95% confidence intervals (CIs). For the binary variables, the odds ratios (ORs) or rate differences (RDs) with 95% CIs were applied for assessment. A *p* < 0.05 was considered to be statistically significantly different.

## Results

### Search Results

**Figure 1:**
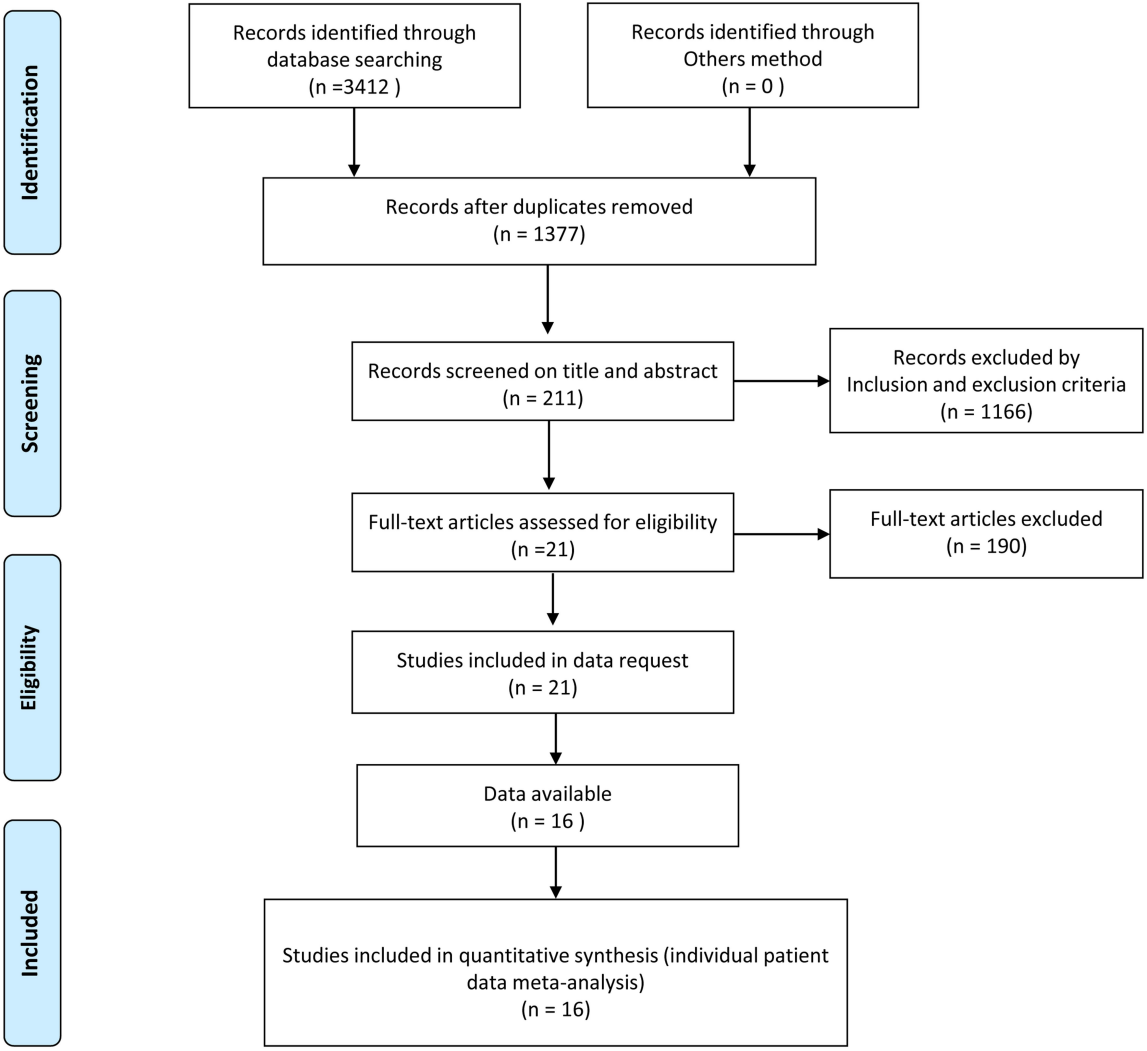
Flowchart of the study selection process.

The initial search resulted in 3,412 English-language full-text studies from the electronic databases of PubMed, Cochrane Library, and EMBASE. Two-thousand-and-thirty-five of the articles were removed as a result of duplicates. One-thousand-one-hundred-and-sixty-six articles were deleted by screening the title and abstract based on the inclusion and exclusion criteria. The remaining 211 studies were used for full-text screening. Finally, 16 articles were selected for our final analysis, including 1003 patients who underwent ETSS, and 992 patients who underwent MTSS. Seven articles directly compared the two interventions for NFPA, Seven for FPA, while three articles compared the two groups for acromegaly. Figure 1 describes this in more detail.

### Methodological Quality Assessment

In this study, the Newcastle-Ottawa Scale (NOS) was adopted to assess the quality of all observational studies with a score range between 0 ~ 9. Two authors (SFG and ZDW) evaluated the quality of the included literature independently. When differences arose in this process, the authors would discuss. All included articles were of high quality, and more details about the quality assessment of the included studies are shown in Table 2.

**Table 2:** The literature quality assessment.

**References**	**Design**	**Newcastle-Ottawa scale (NOS)**			
		**Selection**	**Comparability**	**Exposure**	**Total scores**
Sheehan et al. (13)	Retrospective cohort study	2	2	2	6
Messerer et al. (14)	Retrospective cohort study	4	1	2	7
Dallapiazza et al. (15)	Retrospective cohort study	3	2	3	8
Karppinen et al. (16)	Retrospective cohort study	3	1	2	6
Zaidi et al. (10)	Retrospective cohort study	3	1	3	7
Pledger et al. (25)	Retrospective cohort study	4	2	2	8
Little et al. (17)	Prospective cohort study	4	2	3	9
Haens et al. (26)	Retrospective cohort study	3	1	2	6
Choe et al. (18)	Retrospective cohort study	4	2	2	8
Cheng et al. (19)	Retrospective cohort study	3	2	2	7
Fathalla et al. (20)	Retrospective cohort study	4	1	3	8
Gao et al. (21)	Retrospective cohort study	3	1	3	7
Guo-Dong et al. (22)	Retrospective cohort study	3	1	2	6
Starke et al. (23)	Retrospective cohort study	3	2	2	7
Sarkar et al. (24)	Retrospective cohort study	3	2	2	7
Castaño-Leon et al. (11)	Prospective cohort study	4	2	2	8

*NOS, Newcastle-Ottawa scale*.

### Study Characteristics

In this study, a total of 16 studies (10, 11, 13–26) were included, hailing from Belgium, the USA, India, Finland, France, Korea, Spain, China, and Canada. We enrolled 1003 patients in the ETSS and 992 patients in the MTSS group. Patients with pituitary adenomas were divided into non-functional (775 patients), functional (687 patients), and acromegaly (291 patients) groups. In the NPFA patients, there were seven articles including 994 patients. Of those 994 NFPA participants, 484 belonged to the endoscopic group vs. 510 to the microscopic group. In FPA patients, there were Seven articles including 775 cases. Among those 775 FPA cases, 381 belonged to endoscopic treatment vs. 394 to microscopic treatment. As for the acromegaly group, there were Three articles including 291 participants. More information about the study characteristics is shown in Table 3.

**Table 3:** Characteristics of publication year, country, study type, cases, and gender (Female/Male) in each group for included studies.

**Author**	**Country**	**Years**	**Type of study**	**Sample size (*****n*****)**		**Gender (F/M)**		**Age (mean ± standard)**		**NOS**
				**Endoscopy**	**Microscopy**	**Endoscopy**	**Microscopy**	**Endoscopy**	**Microscopy**	
**Comparison of two intervention for treating non-functioning pituitary adenomas**										
Sheehan et al.	USA	1999	Retrospective	26	44	8/18	13/31	59.2 ± 15.1	57.8 ± 14.9	6
Messerer et al.	France	2011	Retrospective	82	82	35/47	31/51	57.0 (20–82)	56.5 (27–84)	7
Dallapiazza et al.	USA	2014	Retrospective	56	43	19/24	29/27	56.7 ± 16.9	56.2 ± 12.8	8
Karppinen et al.	Finland	2015	Retrospective	41	144	18/23	49/95	58.4 (17–83)	58.5 (16–86)	6
Zaidi et al.	USA	2016	Retrospective	55	80	20/35	30/50	55.9 ± 13.8	59.1 ± 14.6	7
Pledger et al.	USA	2015	Retrospective	47	35	24/23	18/17	52 (32.5–79.5)	54 (27–74)	8
Little et al.	USA	2019	Prospective	177	82	73/104	30/52	58.6 ± 13.3	58.1 ± 14.0	9
**Comparison of two intervention for treating functioning pituitary adenomas6**										
Haens et al.	Belgium	2008	Retrospective	60	60	41/19	16/44	837 (10–70)	35 (10–68)	6
Choe et al.	Korea	2008	Retrospective	12	11	7/5	9/2	47 ± 12	48 ± 10	8
Cheng et al.	China	2011	Retrospective	68	59	37/31	39/20	37.82 (13–69)	33.8 (11–71)	7
Fathalla et al.	Canada	2015	Retrospective	42	23	21/21	16/7	43.2	42.1	8
Gao et al.	China	2016	Retrospective	60	45	34/26	26/19	44.6 (19–75)	48.8 (21–77)	7
Guo-dong et al.	China	2016	Retrospective	100	147	41/59	94/53	43.4 ± 14.0	40.4 ± 14.2	6
Castaño-Leon et al.	Spain	2020	Prospective	39	49	NA	NA	NA	NA	8
**Comparison of two intervention for treating acromegaly**										
Starke et al.	USA	2013	Retrospective	72	41	40/32	21/20	49.2 ± 14.9	47.5 ± 14.2	7
Sarkar et al.	India	2014	Retrospective	66	47	36/30	21/26	37.6 ± 10.8	38.7 ± 12.2	7
Fathalla et al.	Canada	2015	Retrospective	42	23	21/21	16/7	43.2	42.1	8

*NA, not available; F, female; M, male; NOS, Newcastle-Ottawa Scale*.

### Outcomes

#### Analyzed Items

The items analyzed in this study were: (1) the GTR, based on postoperative imaging confirming tumor absence; (2) the time of operation and length of stay for two surgery; and (3) postoperative complications (visual improvement, CSF leak, diabetes insipidus, hypothyroidism, hypocortisolism, meningitis, hematoma, hypopituitarism, and mortality). More information is shown in Table 4.

**Table 4:** The postoperative outcomes of this meta-analysis.

**Outcomes**	**Studies numbers**	**Groups size**		**Overall effect**			**Heterogeneity**	
		**Endoscopic**	**Microscopic**	**Effect estimate**	**95% CI**	***P*-value**	***I*^**2**^(%)**	***P*-value**
**Comparison of two interventions for treating non-functioning pituitary adenomas**								
Gross-total resection	5	250	383	OR, 1.655	1.131, 2.421	**0.010**	32.3%	0.206
CSF leak	7	472	510	RD, −0.010	−0.041, 0.020	0.506	0.0%	0.916
Diabetes insipidus	6	339	559	OR, 1.033	0.610, 1.751	0.903	32.5%	0.192
Visual improvement	2	69	93	OR, 3.636	0.634, 20.849	0.147	0.0%	0.366
Meningitis	4	355	388	RD, −0.004	−0.024, 0.015	0.653	0.0%	0.942
Hematoma	5	381	432	OR, 0.788	0.286, 2.169	0.645	35.5%	0.185
Hypopituitarism	4	171	244	OR, 0.753	0.433, 1.309	0.315	13.2%	0.327
Hypothyroidism	2	152	103	OR, 0.582	0.269, 1.259	0.169	0.0%	0.773
Hypocortisolism	3	246	182	OR, 0.640	0.142, 2.890	0.562	82.7%	0.003
Total mortality	2	259	164	RD, −0.001	−0.020, 0.019	0.958	0.0%	0.960
Length of Stay	3	288	205	WMD, 0.112	−0.791, 1.014	0.808	61.2%	0.076
**Comparison of two interventions for treating functioning pituitary adenomas**								
Gross–total resection	5	229	244	OR, 2.033	1.335, 3.096	**0.001**	17.6%	0.302
CSF leak	6	342	345	OR, 1.054	0.535, 2.076	0.880	0.0%	0.445
Diabetes insipidus	6	341	237	RD, −0.136	−0.319, 0.047	0.145	96.7%	0.000
Visual improvement	3	71	54	OR, 2.461	1.109, 5.459	**0.027**	25.6%	0.261
Meningitis	4	232	263	OR, 0.195	0.041, 1.923	**0.039**	0.0%	0.998
Hematoma	2	112	158	RD, 0.015	−0.023, 0.053	0.440	0.0%	0.838
Hypopituitarism	5	282	285	OR, 0.675	0.299, 1.521	0.343	48.4%	0.101
Total mortality	2	120	105	RD, 0.000	−0.025, 0.025	1.000	0.0%	1.000
Length of Stay	3	228	251	WMD, −1.284	−3.656, 1.089	0.289	95%	0.000
Operation time	4	325	336	WMD, 4.022	−53.674, 61.719	0.891	99.0%	0.000
**Comparison of two interventions for treating acromegaly**								
CSF leak	3	181	111	OR, 0.581	0.163, 2.079	0.404	0.0%	0.791
Diabetes insipidus	3	179	111	OR, 0.905	0.203, 4.029	0.896	68.4%	0.042
Hypopituitarism	2	108	70	OR, 1.214	0.531, 2.77	0.646	0.0%	0.368
Hypothyroidism	2	136	86	OR, 0.576	0.228, 1.457	0.244	0.0%	0.560
Hypocortisolism	2	132	87	OR, 0.703	0.111, 4.476	0.709	77.8%	0.034

*CIs, confidence intervals; RD, rate difference; OR, odds ratio; CSF, Cerebrospinal fluid. Bold values means P < 0.05*.

### Comparison of Two Interventions for Treating Non-functioning Pituitary Adenomas

#### Gross-Total Resection (GTR)

**Figure 2:**
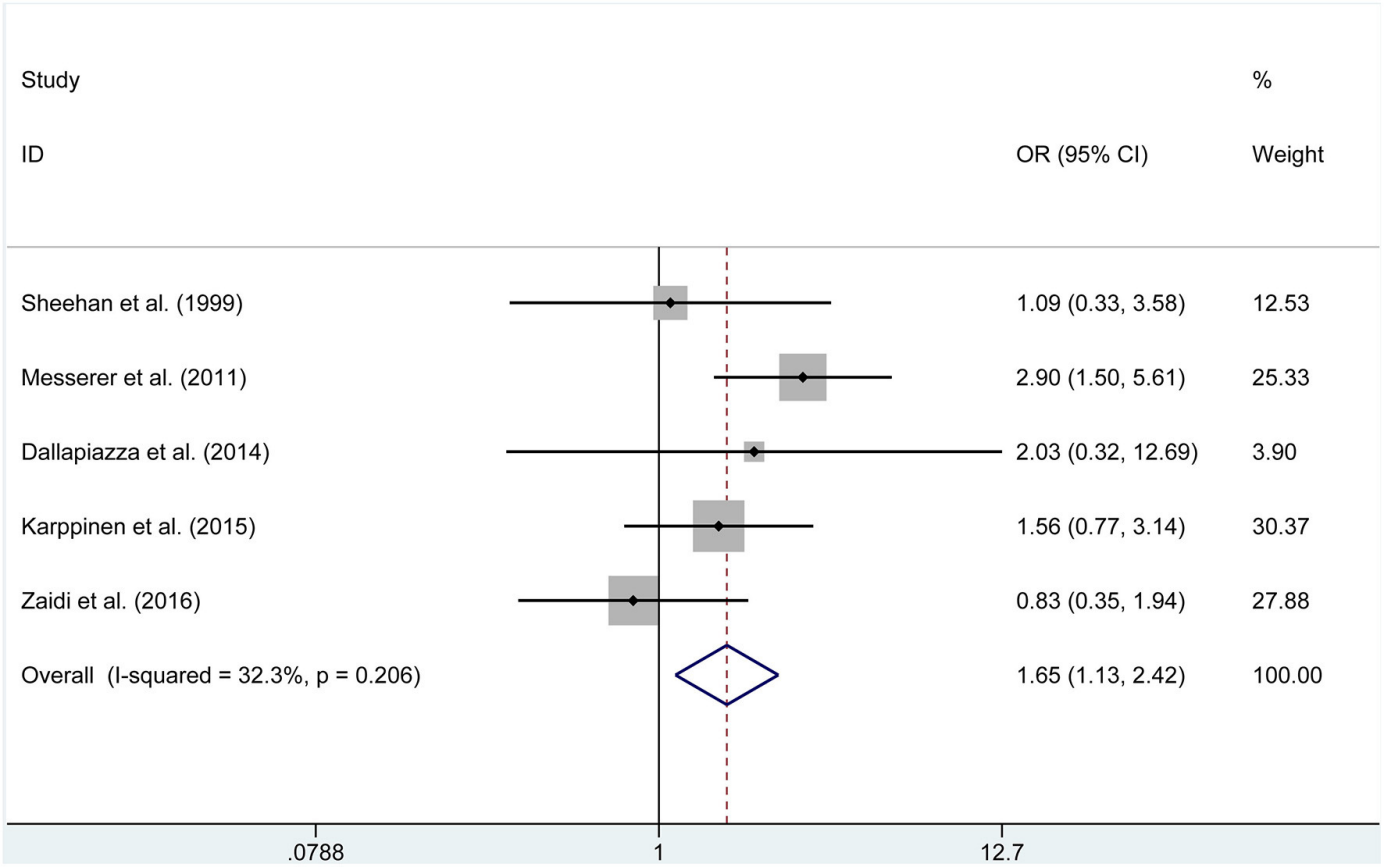
Forest plot on gross-total resection with ETSS vs. MTSS for NFPA.

We adopted meta-analytical techniques to assess the incidence of postoperative GTR. Reviewing the data of the included studies, five publications (250 endoscopic and 383 microscopic) reported on the postoperative GTR. We selected the fixed effects model because the heterogeneity was not significantly different (*P* = 0.206, *I*^2^ =32.3%). The ETSS group was related to a higher incidence of postoperative GTR in NFAP participants (OR = 1.655, 95% CI 1.131–2.421, *P* = 0.010, Figure 2).

#### Length of Stay

Three studies (288 endoscopic, 205 microscopic) reported data on length of stay. We found that the difference between the two surgeries was not statistically significant (WMD = 0.112, 95% CI −0.791 to 1.014, *P* = 0.808).

#### Postoperative Complications

**Figure 3:**
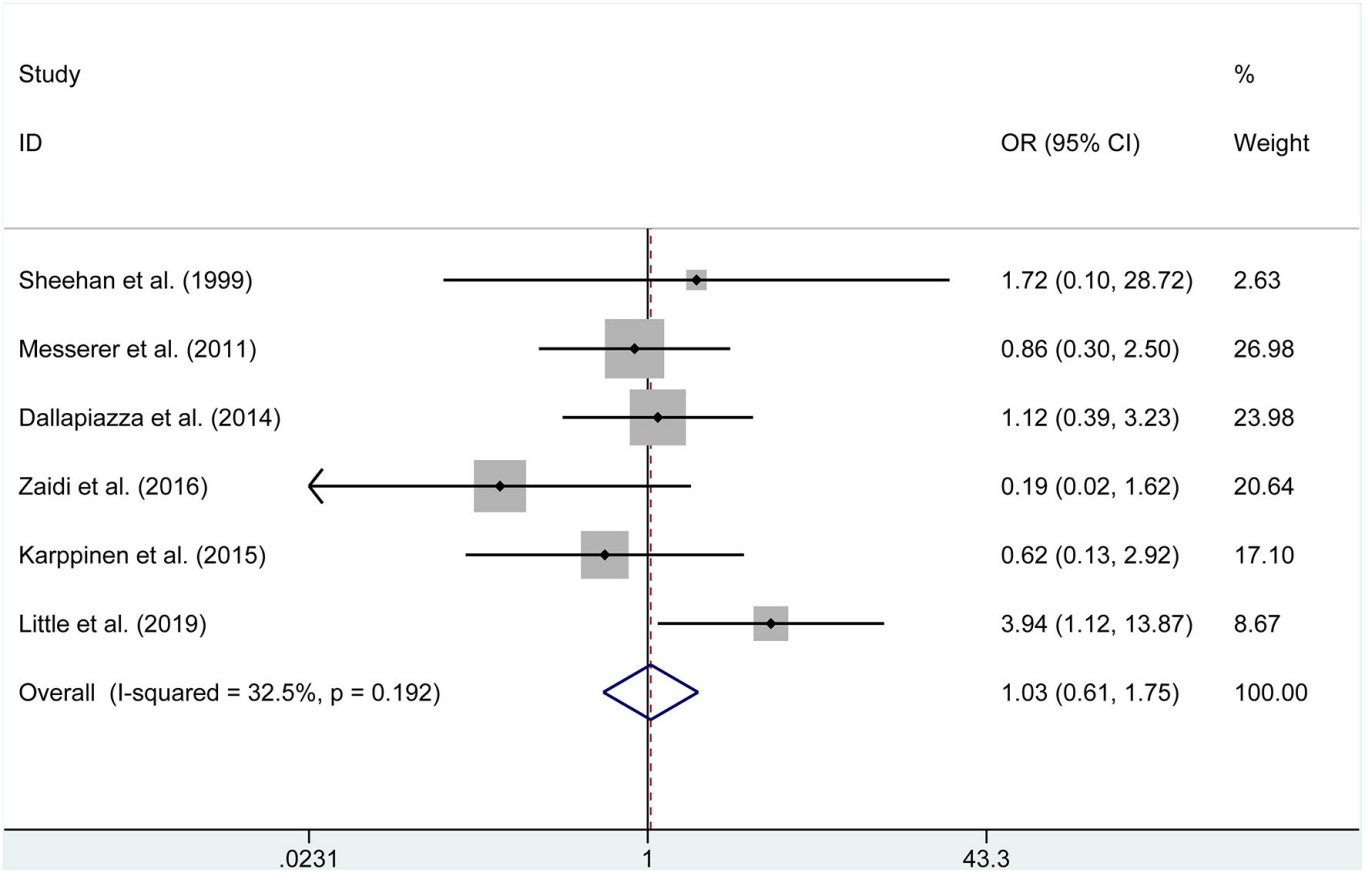
Forest plot on diabetes insipidus with ETSS vs. MTSS for NFPA.

**Figure 4:**
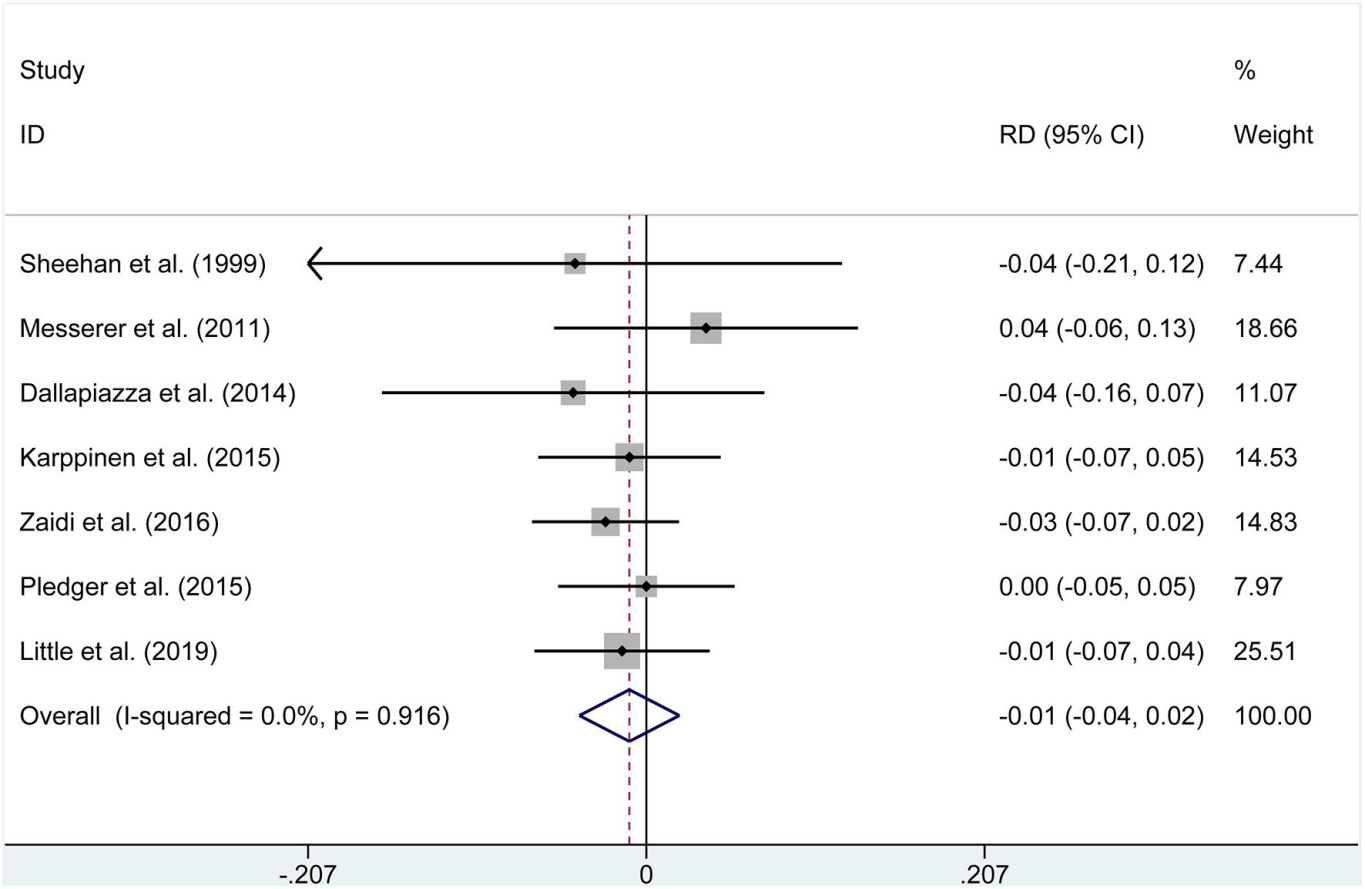
Forest plot on cerebrospinal fluid leak with ETSS vs. MTSS for NFPA.

The pooled estimates of the overall proportions showed no significant difference in the incidence of visual improvement (OR = 3.636, *P* = 0.147), diabetes insipidus (OR = 1.033, *P* = 0.903, Figure 3), hypocortisolism (OR = 0.640, *P* = 0.562), hematoma (OR = 0.788, *P* = 0.645), CSF leak (RD = −0.01, *P* = 0.506, Figure 4), hypopituitarism (OR = 0.753, *P* = 0.315), meningitis (RD = −0.004, *P* = 0.653), hypothyroidism (OR = 0.582, *P* = 0.169), and mortality (RD = −0.001, *P* = 0.958) according to the data of seven studies.

### Comparison of Two Interventions for Treating Functioning Pituitary Adenomas

#### Gross-Total Resection (GTR)

**Figure 5:**
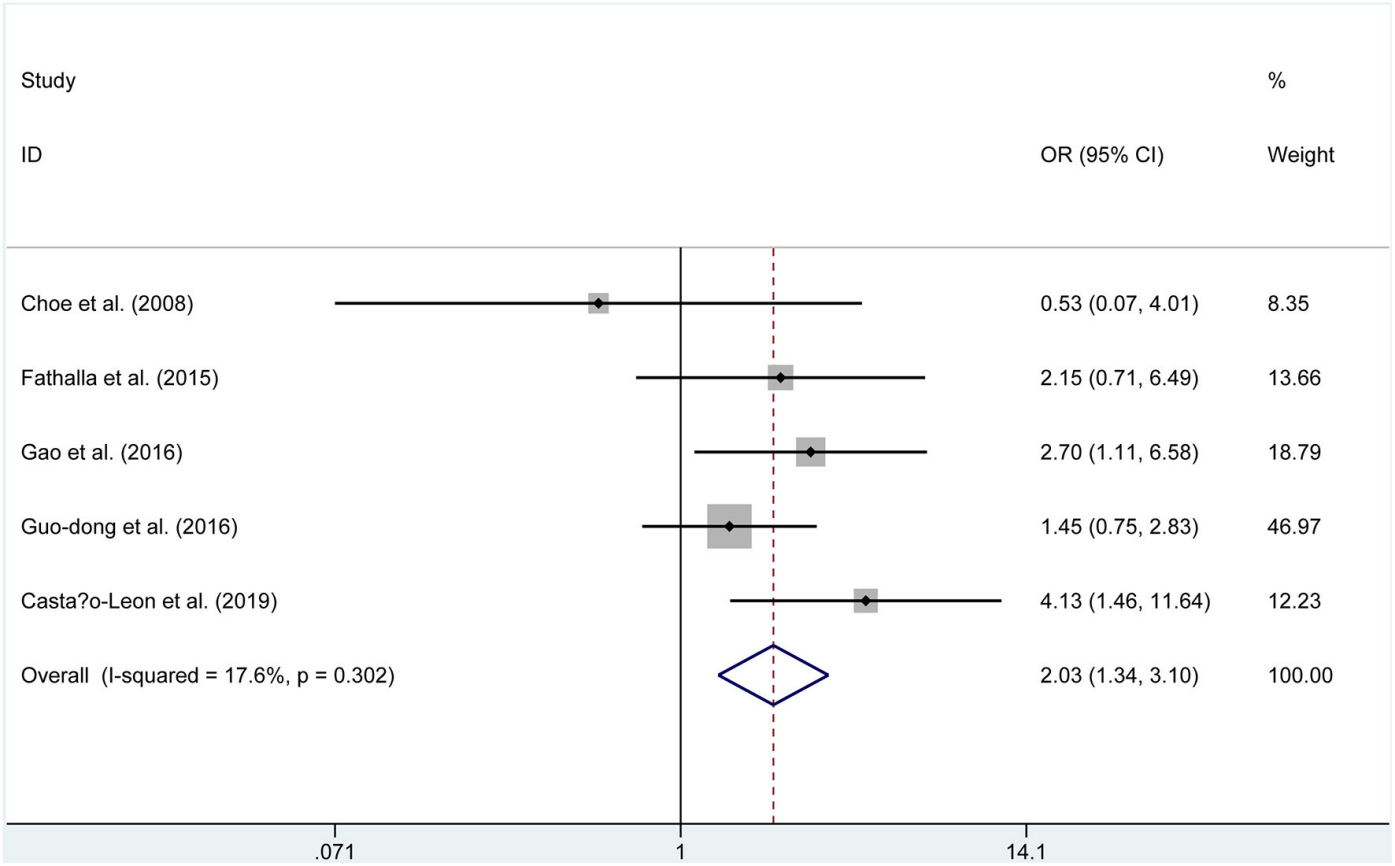
Forest plot on gross-total resection with ETSS vs. MTSS for FPA.

Five publications (229 endoscopic, 244 microscopic) reported on the postoperative GTR. A fixed-effects model was used to assess this, due to no significant heterogeneity (*P* = 0.302, *I*^2^ = 17.6%). The GTR rate was 73.8% in the endoscopic group and 62.3% in the microscopic group. The pooled evaluation of the overall proportions demonstrated that a significantly higher rate of GTR appeared in the ETSS group (OR = 2.033, 95% CI 1.335–3.096, *P* = 0.001, Figure 5).

#### Time of Operation and Length of Stay

Three studies (228 endoscopic, 251 microscopic) reported data on length of stay. We found that the difference was not statistically significant between the two groups (WMD = −1.284, 95% CI −3.656 to 1.089, *P* = 0.289). Similarly, the results showed that there was no significant difference found in the operation time (WMD = 4.022, 95% CI −53.674 to 61.719, *P* = 0.891).

#### Postoperative Complications

**Figure 6:**
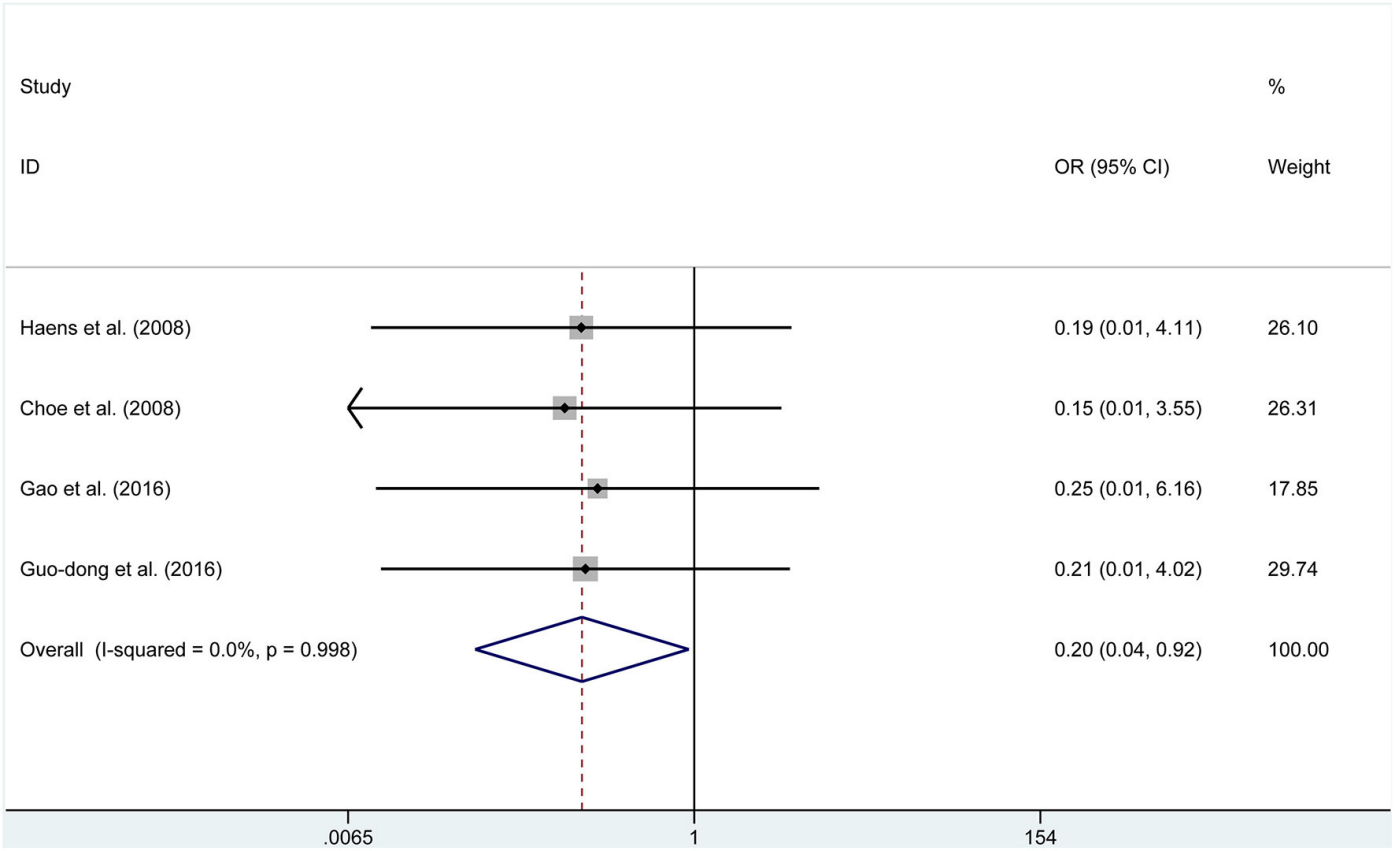
Forest plot on meningitis with ETSS vs. MTSS for FPA.

**Figure 7:**
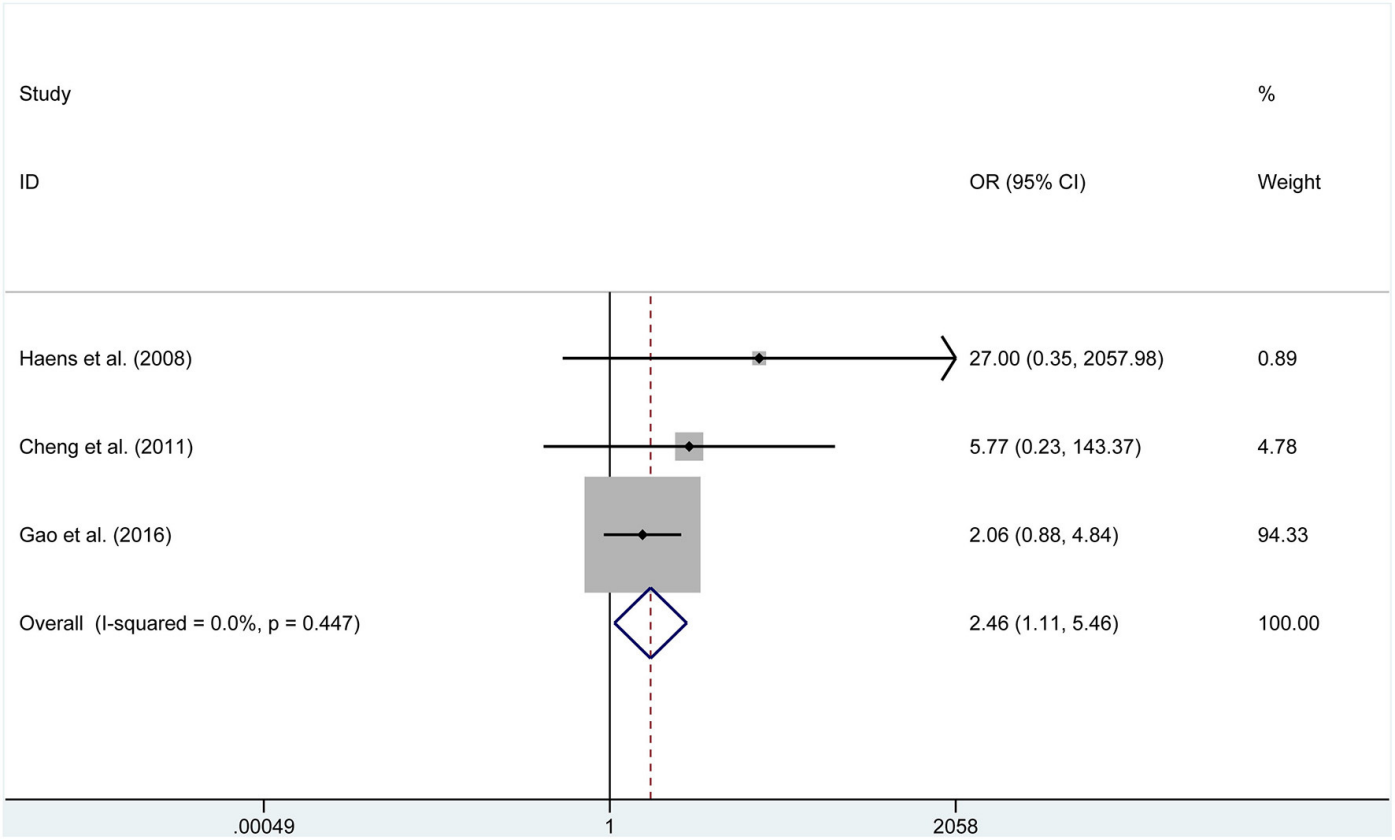
Forest plot on visual improvement with ETSS vs. MTSS for FPA.

Four studies reported data on meningitis. No significant heterogeneity was found, then the fixed effects model was selected (*P* = 0.998, *I*^2^ = 0%). We found that endoscopic treatment was related to lower meningitis rates than in the microscopic group (OR = 0.195, 95% CI 0.041–1.923, *P* = 0.039, Figure 6). Furthermore, endoscopic treatment had a higher incidence of visual improvement (OR = 2.461, 95% CI 1.109–5.459, *P* = 0.027, Figure 7). However, according to the data of six studies, a significant difference was not found between the two groups for diabetes insipidus (RD = −0.136, *P* = 0.145), hypocortisolism (OR = 0.675, *P* = 0.343), hematoma (RD = 0.015, *P* = 0.440), CSF leak (OR = 1.054, *P* = 0.880), and mortality (RD = 0.000, *P* = 1.000).

### Comparison of Two Interventions for Treating Acromegaly

#### Postoperative Complications

The pooled estimates of the overall proportions showed that the incidence of CSF leak (OR = 0.581, *P* = 0.404), hypopituitarism (OR = 1.214, *P* = 0.646), diabetes insipidus (OR = 0.905, *P* = 0.896), hypothyroidism (OR = 0.576, *P* = 0.244), and hypocortisolism (OR = 0.703, *P* = 0.709) was not significantly different between the two groups based on the data of three studies.

## Discussion

### Summarizing the Objectives and Main Findings

The current common operations for pituitary adenomas are ETSS and MTSS. Although the merits and disadvantages of ETSS vs. MTSS for treating pituitary adenoma have been assessed previously, the comparisons of two interventions, specifically on NFPA and FPA, have never been comprehensive and systematically performed before. Previous meta-analyses do not include a purely comparative study to evaluate the efficacy and safety between the two interventions, resulting in their ability to provide certain evidence for intervention remaining controversial. Thus, we conducted this meta-analysis to explore whether there were any differences in the risk of postoperative complications between the two groups in cases with NFPA or FPA. This quantitative analysis included 1995 patients with pituitary adenoma assessed in 16 studies, and this pooled data showed that FPA or NFPA patients treated by ETSS, had a higher incidence rate of GTR.

### MTSS Had a Higher Rate of GTR in Patients With NFPA or FPA

In this meta-analysis, the pooled statistic revealed that the NFPA participants treated with endoscopes had a higher incidence of GTR (OR = 1.655, 95% CI 1.131–2.421, *P* = 0.010). Likewise, a meta-analysis conducted by Gao et al. (27) found that the higher incidence of GTR appeared to be in patients treated with ETSS rather than those treated by MTSS (*P* = 0.0001), consistent with the study conducted by Yu et al. (28) (*P* < 0.001) Similarly, we also revealed that the proportion of GTR was also relatively higher in FPA cases treated by ETSS compared to patients treated by MTSS (OR = 2.033, 95% CI 1.335–3.096, *P* = 0.001). Parasellar extension accounted for most resection outcomes, with a panoramic view where endoscopy provides a wider and superior route to parasellar and suprasellar compartments, contributing to the higher rates of GTR (29). In brief, the endoscope is particularly useful in obtaining a panoramic view of the surrounding structures through the use of angled endoscopes.

### ETSS Resulted in a Lower Incidence of Meningitis and a High Incidence of Visual Improvement

Beyond what is described above, the lower rates of meningitis in patients with FPA undergoing ETSS was shown by the pooled data (OR = 0.195, 95% CI 0.041–1.923, *P* = 0.039). The origin behind the effect might be from the superiorities of ETSS, such as a shorter operation time. Moreover, there was a higher percentage of patients with FPA treated with ETSS who gained visual improvement (OR = 2.461, 95% CI 1.109–5.459, *P* = 0.027). ETSS has been proven to be more capable of exploring sella turcica with a panoramic view, which increases the success incidence and fully eliminates compression on optic chiasma due to sellar region lesions. Concerning patients with acromegaly, ETSS and MTSS demonstrated no statistically significant difference in controlling postoperative complications, which might be caused by the shortage of data extracted from only three included studies. With an increase in highly-qualified studies related to surgical treatment of acromegaly reported in the future, results of significance may yet be revealed.

### ETSS Did Not Lead to the Higher Rates of Postoperative Complications Such as CSF Leak and Diabetes Insipidus

There was no significant difference in the CSF leak and diabetes insipidus between the two modalities, irrespective of being NFPA or FPA patients. Reconstructive procedures following resection should be acknowledged as an essential factor for the risk of a postoperative leak of CSF (30). Theoretically, reconstruction performed with the endoscope, offering wider visualization, enjoyed a higher success rate. This would reduce the incidence of CSF leakage for patients treated by MTSS to some extent. Whereas, in this study, the discrepant incidence of postoperative CSF leaks was of no statistical significance. The main cause for this might be that elevated exposure during ETSS would result in more aggressive surgical exploration, leading to an increased rate of postoperative CSF leaks. Besides that, a learning curve (31, 32) was expected owing to the fact that ETSS was an updated technique, while it was impossible to incorporate a surgeon's level of experience. Thus, although actual complications might be different between ETSS and MTSS, the differences may be neutralized by the above factors. Additionally, a non-significant correlation exists between the lower rates of diabetes insipidus and ETSS when compared to MTSS, despite ETSS acquiring a higher rate of GTR. We suspect that the tumor type might play a key role in this effect (29), whereas no stratified results were described for most included trials to help us conduct a subgroup analysis.

### Study Limitations

This study had some limitations; (I) a great many of the related comparative articles were excluded from this articles due to the fact that various pituitary adenomas subtypes were involved and where specific information about NFPA or FPA could not be obtained; (II) this study assessed all endpoints with different follow-ups between the two groups, which could cause a bias; (III) although this study used a rigorous search strategy to identify all relevant studies, a small number of studies might have been overlooked; and (IV) microadenomas are different than invasive, cavernous sinus extended macroadenoma, and we were unable to perform a subgroup analysis based on adenoma size and Hardy-Wilson classification, due to the limited data.

## Conclusion

Based on current evidence, NFPA patients treated by endoscopy were had higher rates of GTR; FPA patients treated by ETSS had a higher rate of visual improvement and GTR, as well as a lower rate of meningitis.

## Data Availability Statement

The original contributions presented in the study are included in the article/supplementary material, further inquiries can be directed to the corresponding author/s.

## Author Contributions

SG and XL designed the study, acquired the data, drafted the article, and analyzed and interpreted the data. ZW and XK revised the article critically for important intellectual content together. All authors approved the version to be published.

## Conflict of Interest

The authors declare that the research was conducted in the absence of any commercial or financial relationships that could be construed as a potential conflict of interest.

## Abbreviations

MTSS: microscopic transsphenoidal pituitary surgery
GTR: Gross-total resection
NFPA: non-functioning Pituitary Adenomas
FPA: Functioning Pituitary Adenomas
ETSS: endoscopic transsphenoidal pituitary surgery
Cis: confidence intervals
WMD: weighted mean differences
CSF: cerebrospinal fluid
RDs: rate differences
ORs: odds ratios
PRISMA: Preferred Reporting Items for Systematic Reviews and Meta-Analyses.
